# Dataset and GIS toolbox for modeling potential tree belt functions

**DOI:** 10.1016/j.dib.2018.08.005

**Published:** 2018-08-08

**Authors:** Maciej M. Nowak, Katarzyna Pędziwiatr

**Affiliations:** Laboratory of Biological Spatial Information, Faculty of Biology, Adam Mickiewicz University in Poznań, ul. Umultowska 89, 61-614 Poznań, Poland

**Keywords:** Greenways planning, Linear woody features, Open source toolbox

## Abstract

The dataset and toolbox presented in this article are related to the research article entitled "Modeling potential tree belt functions in rural landscapes using a new GIS tool" (Nowak and Pędziwiatr, 2018) [1]. The dataset (spatially referenced) is used as input for all the five analysis modules described in the related article. The dataset contains vector and raster data, which cover north-west part of the Gen. Dezydery Chłapowski Landscape Park in Poland. Moreover, the present work shows the result (a vector layer) of using the TBM toolbox within ArcGIS (ESRI) software. The Tree Belt Modeling (TBM toolbox) code and internal structure can be fully accessed, and are available for further modification and incorporation of tools. The toolbox is available for download as supplementary material to this online article.

**Specifications Table**

**Table:** 

Subject area	*Environmental science*
More specific subject area	*Green infrastructure management*
Type of data	*Vector and raster spatial data, ArcGIS toolbox, figures*
How data was acquired	*From* Head Office of Geodesy and Cartography in Warsaw, *GIS-based processing*
Data format	*Raw, Processed and Analyzed*
Experimental factors	*The new GIS toolbox is developed by using ArcGIS 10.5 software, Python programming language and Model Builder toolbox for ArcGIS (ESRI).*
Experimental features	*vector data (ESRI shapefile format): input polylines layer (including cadastral data, roads and streams) and polygon layer (land cover), output polylines layer (the result obtained from the new GIS toolbox); raster data (Geo Tagged Image File Format): the digital elevation model (DEM); toolbox structure (tbx file format): Python scripts and Model Builder structure;* All these data are defined in the polish spatial reference system PL 1992.
Data source location	*Poland, north-west part of the Gen. Dezydery Chłapowski Landscape Park (centroid point: 16,76° E, 52,08° N)*
Data accessibility	*All data are available with this article*
Related research article	*M.M. Nowak, K. Pędziwiatr, Modeling potential tree belt functions in rural landscapes using a new GIS tool, Journal of Environmental Management, 217 (2018) 315–326. doi: 10.1016/j.jenvman.2018.03.118*

**Value of the data**

● The input dataset could be used to assess the degree of potential tree belt function availability in the north-west part of the Gen. Dezydery Chłapowski Landscape Park in Poland.
● The data can be used for testing the TBM toolbox by a potential user and serve as a reference for the structure of any other dataset to be used in the toolbox.
● Open access to the TBM toolbox internal structure allows researchers to further develop it by adding new parameters, modules or incorporating the TBM toolbox into other GIS tools and software.

## 1 Data

This article presents a dataset (spatially referenced) over the Gen. Dezydery Chłapowski Landscape Park in Poland. It includes input data acquired from Head Office of Geodesy and Cartography in Warsaw and output data received from the Tree Belt Modeling (TBM) toolbox processing. The first part of the dataset contains vector layers (format: ESRI shapefile): polylines layer (including cadastral data, roads and streams) and polygon layer (land cover). The digital elevation model (Geo Tagged Image File Format) is also added to the first part. The second part contains the output vector layers obtained from all the five analysis modules described in the related article. The result of the TBM final analysis (elementary, semi-integrated and integrated level of the tree belt functions availability [1]) is also added to the dataset. All these data are defined in the polish spatial reference system PL 1992. The input dataset was used for developing and testing the TBM toolbox incorporated to ArcGIS software. This toolset (tbx file for three versions of ArcGIS - 10.2, 10.3, 10.5) is available to download with this article. The toolbox was developed based on the editable Model Builder (MB; ArcGIS, ESRI) structure and partly on Python scripts.

## 2 Experimental design, materials and methods

The TBM toolbox was developed using spatial data from the north-west part of the Gen. Dezydery Chłapowski Landscape Park (protected area) situated in western Poland. This part of the park was selected based on the assessment of the density of linear structures (watercourses, drainage ditches, roads, and plot boundaries) over the entire park. To reliably test the TBM tools, the area with the highest density of linear structures was selected. It was assumed that the dense network of linear objects concentrated within a small area is appropriate for checking the efficiency of the TBM tools because of higher probability of detection of possibly untypically distributed linear structures. The selected part of the park covers 5460.95 ha of agricultural area (90.93%) and 495.19 ha of forested area (9.07%). The total length of linear objects as potential places for linear woody features is 1023.12 km.

The presented toolset was based on the Model Builder (MB; ArcGIS, ESRI). MB facilitates the development of spatial models without the necessity of combining the typical developing software programs and advanced programming skills of a user [2]. Moreover, MB enables a full automation of a complex data processing using existing ArcGIS tools. The MB formula gives the opportunity for editing geoprocessing schemes. In addition, the TBM toolbox contains Python scripts. This open source programming language allows to develop new software, tools and combine existing components in a computing environment, including the GIS software [3]. It should be emphasized that the ArcGIS structure makes it more possible to compile many Python scripts and MB tools into one toolset that can be easily distributed and included in other software. This aspect makes Python scripts available to both programmers and GIS users. The use of scripts and MB enables their integration with other GIS tools [4], improves computer processing efficiency and facilitates their implementation [5].

**Fig. 1:**
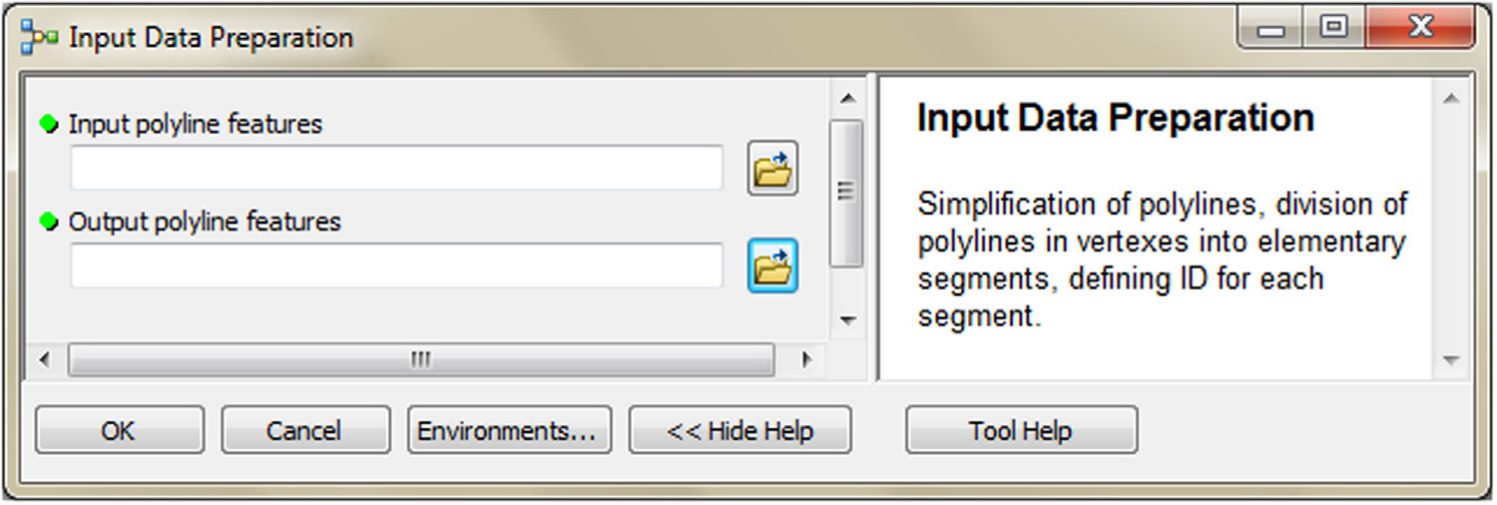
User interface of the input data preparation tool.

**Fig. 2:**
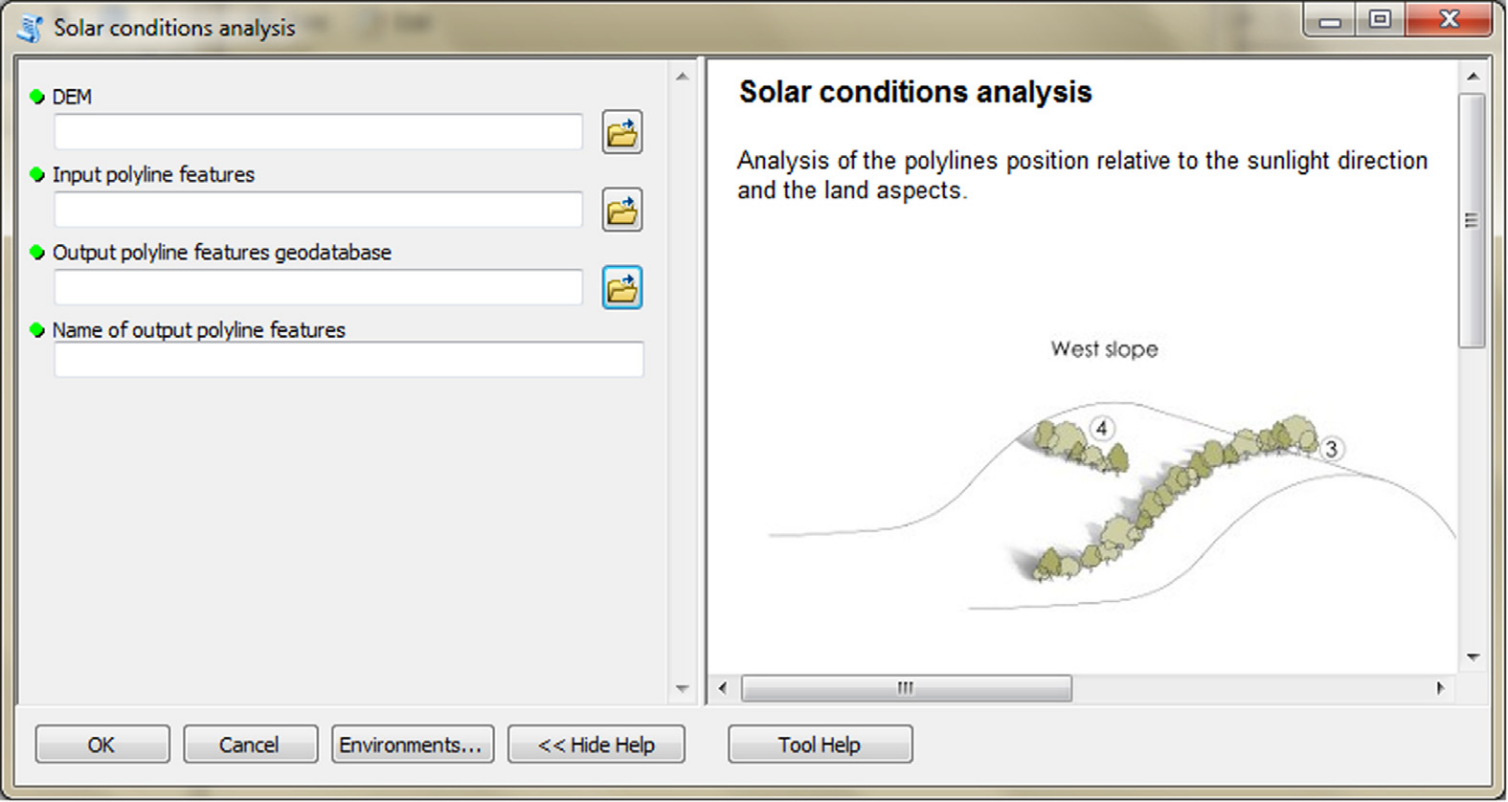
User interface of the solar conditions analysis tool.

**Fig. 3:**
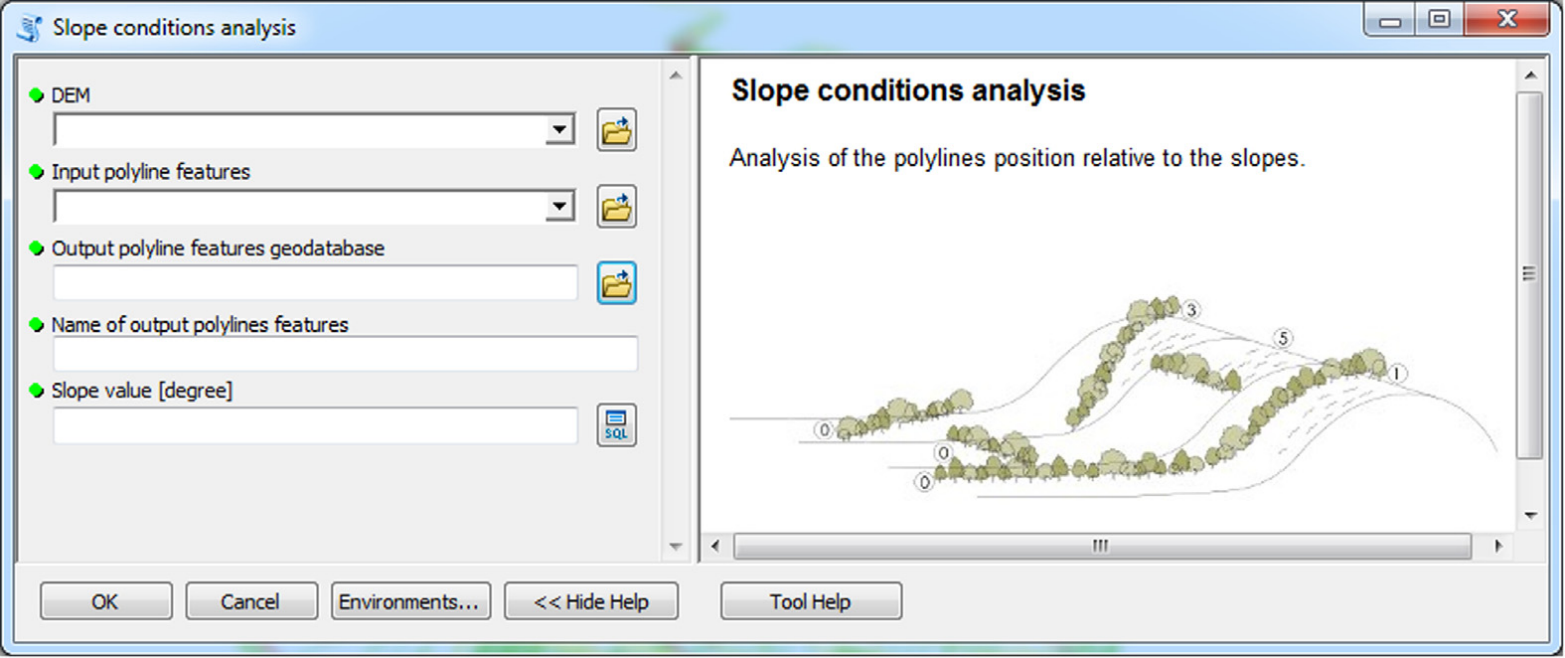
User interface of the slope conditions analysis tool.

**Fig. 4:**
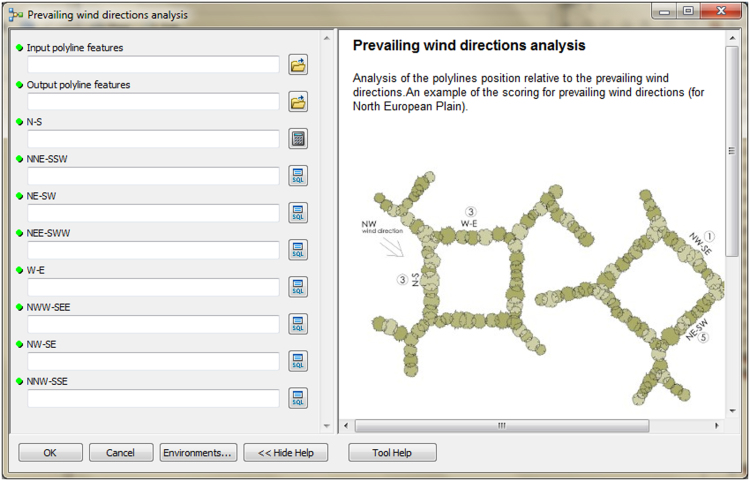
User interface of the prevailing wind directions analysis tool.

**Fig. 5:**
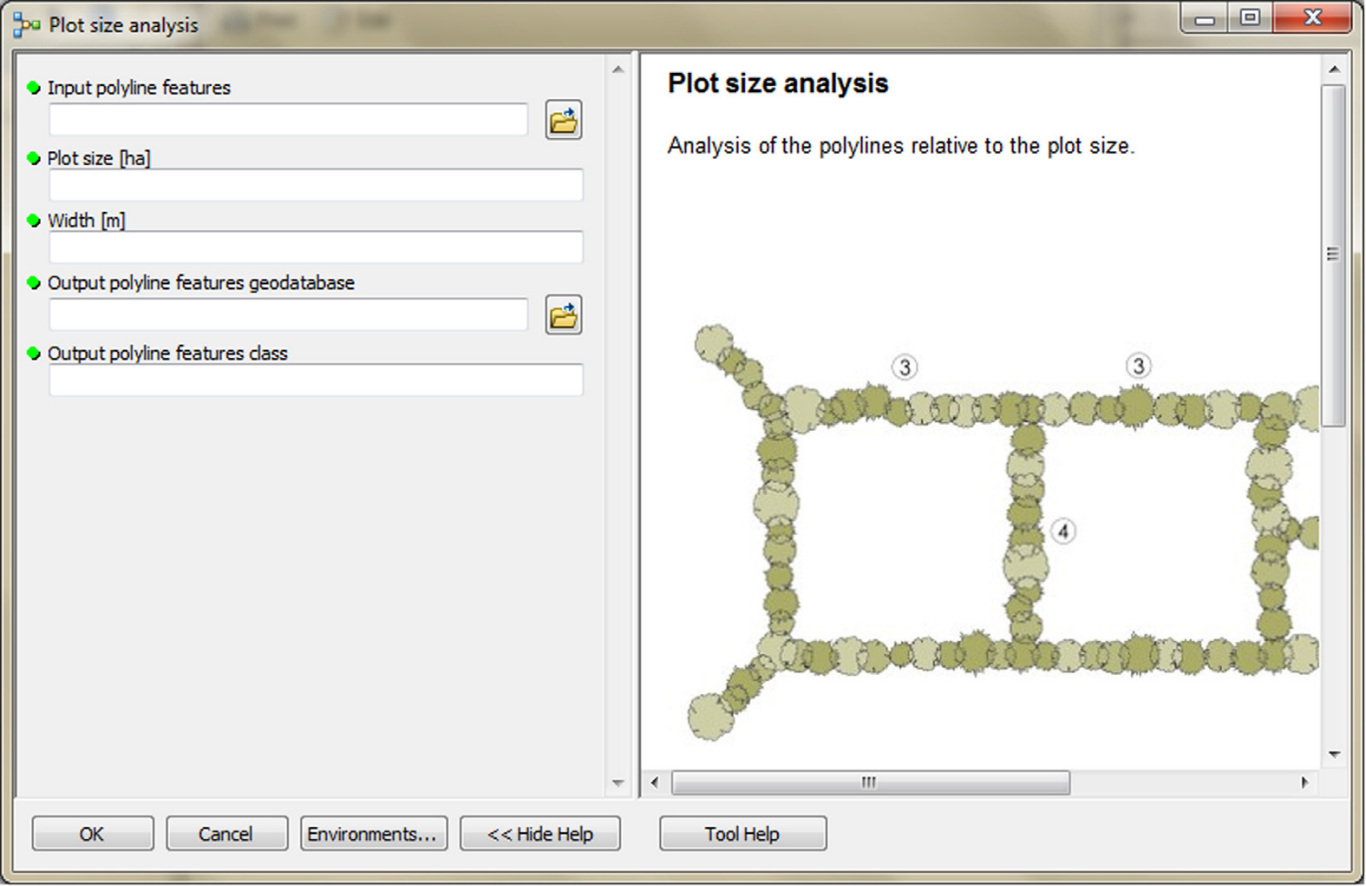
User interface of the plot size analysis tool.

**Fig. 6:**
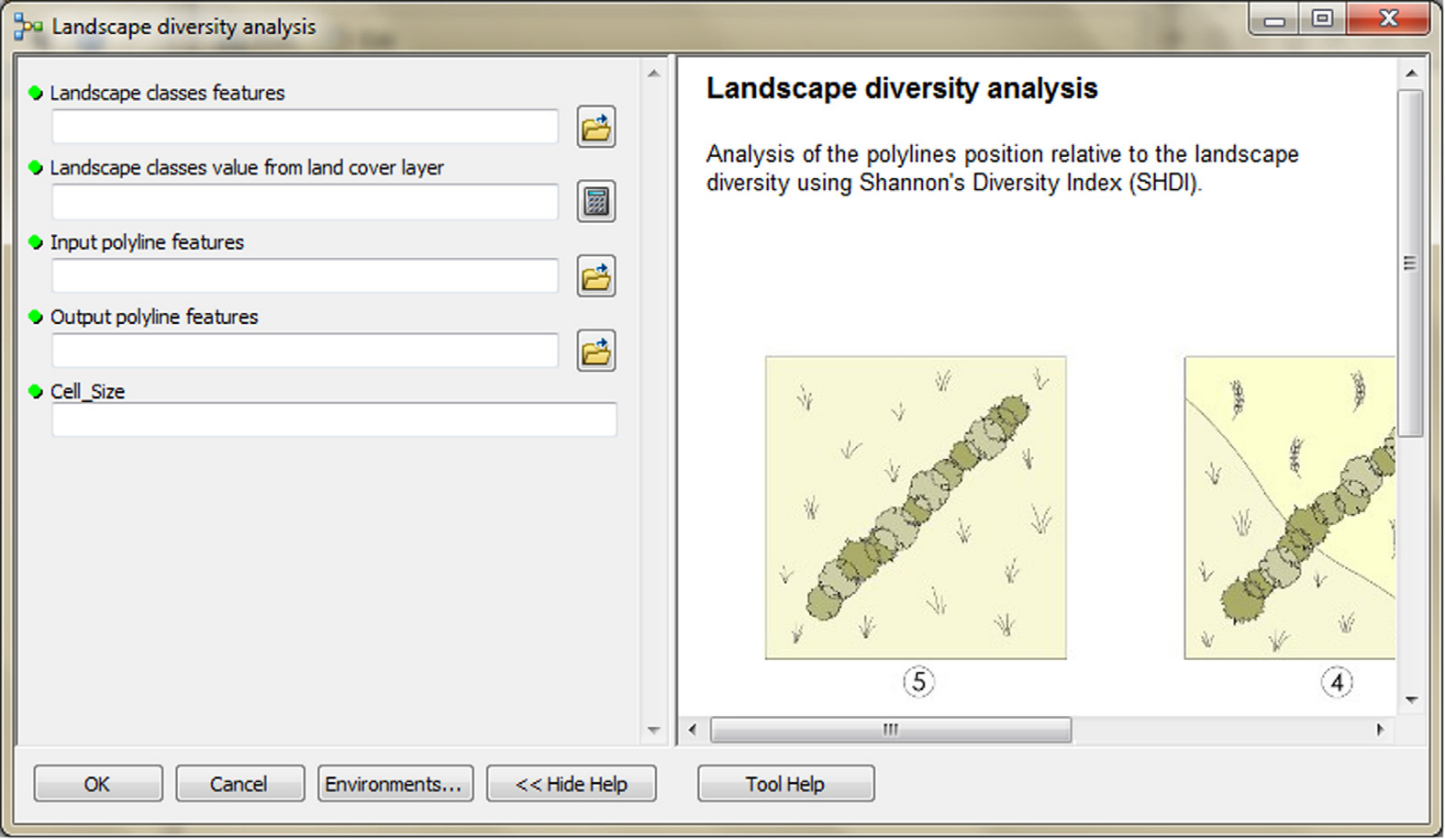
User interface of the landscape diversity analysis tool.

**Fig. 7:**
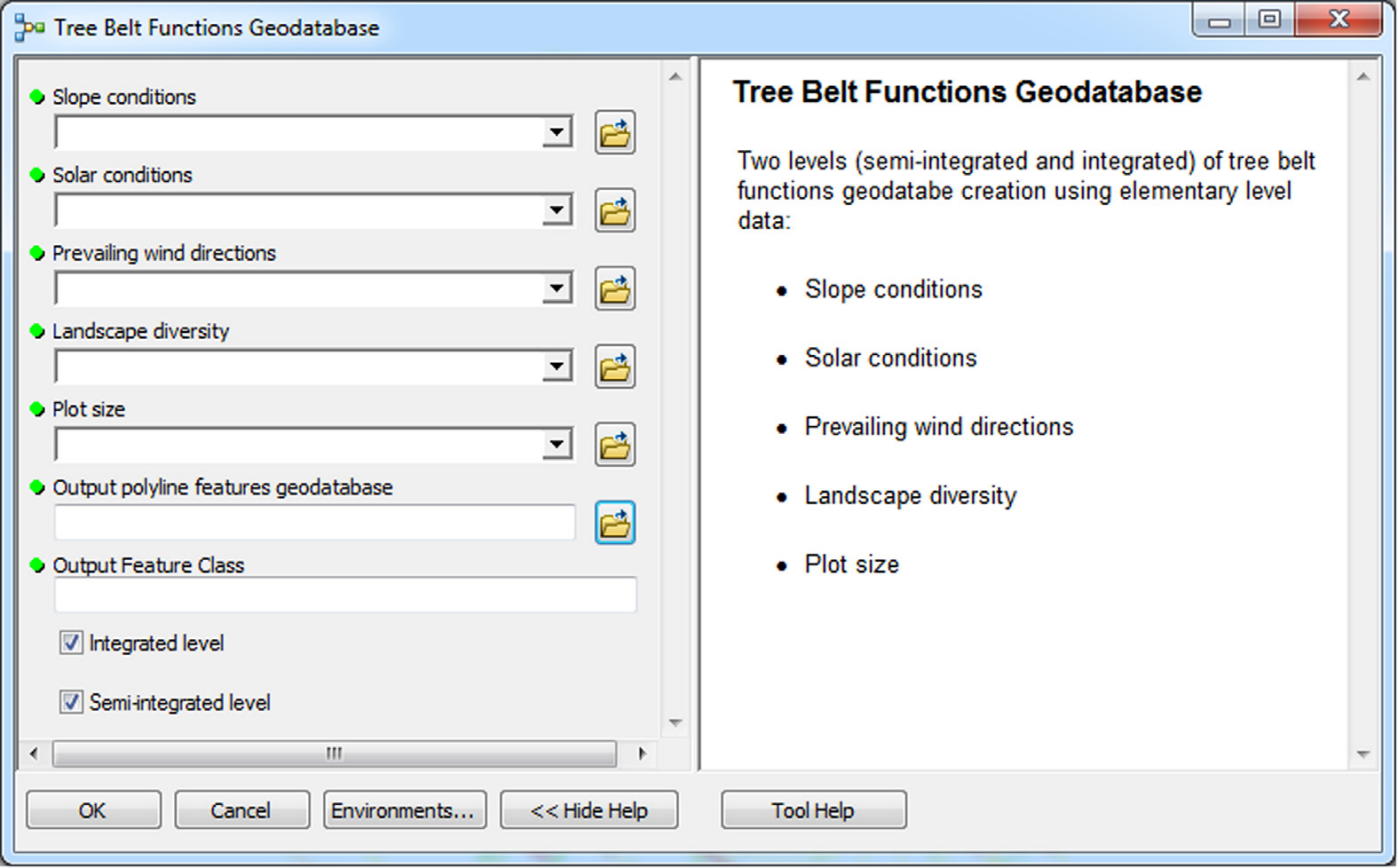
User interface of the Tree Belt Functions Geodatabase tool.

The input data preparation tool from the first part of TBM toolbox (Fig. 1) was used for creating the input dataset (vector layer: all_lines). This layer was performed on the basis of the digital topographic database (accuracy: 1:10 000), the digital cadastral database (accuracy: 1:5 000) and the digital hydrographic database (accuracy: 1:10 000). The second vector layer (land_cover.shp) was obtained from digital topographic database using the *Select by attributes* tool and the *Field calculator* tool in ArcMap. To prepare the Digital Elevation Model (DEM) on the basis of Airborne Laser Scanning data (density of point cloud: 4–6 points/m²), the LAS dataset toolbar and the *LAS dataset to raster* tool (Conversion tools in ArcMap) were used. In the second part of the TBM toolbox, Python scripts were developed for the solar conditions analysis tool (Fig. 2) and the slope conditions analysis tool (Fig. 3). In these two cases, there was a need to develop new geoprocessing parameters. The three other tools in this part of the toolbox present MB structures solely based on existing ArcGIS tools: the prevailing wind direction analysis (Fig. 4), the plot size analysis tool (Fig. 5) and the landscape diversity analysis tool (Fig. 6). The third part of the TBM toolbox is the tree belt functions geodatabase tool (Fig. 7) used for performing the semi-integrated and the integrated level of function availability. The TBM toolbox has been saved in the tbx format. Its structure allows full editing and modifying of the MB construction or Python scripts adding new analytical parameters or transferring selected parts into other GIS tools.
